# Inattentional blindness for a gun during a simulated police vehicle stop

**DOI:** 10.1186/s41235-017-0074-3

**Published:** 2017-09-20

**Authors:** Daniel J. Simons, Michael D. Schlosser

**Affiliations:** 1 Department of Psychology, University of Illinois at Urbana-Champaign, 603 E. Daniel Street, Champaign, IL 61820 USA; 2 University of Illinois at Urbana-Champaign, Police Training Institute, Champaign, IL USA

**Keywords:** Attention, Perception, Inattentional blindness, Awareness, Detection, Expertise, Consciousness

## Abstract

People often fail to notice unexpected objects and events when they are focusing attention on something else. Most studies of this “inattentional blindness” use unexpected objects that are irrelevant to the primary task and to the participant (e.g., gorillas in basketball games or colored shapes in computerized tracking tasks). Although a few studies have examined noticing rates for personally relevant or task-relevant unexpected objects, few have done so in a real-world context with objects that represent a direct threat to the participant. In this study, police academy trainees (n = 100) and experienced police officers (n = 75) engaged in a simulated vehicle traffic stop in which they approached a vehicle to issue a warning or citation for running a stop sign. The driver was either passive and cooperative or agitated and hostile when complying with the officer’s instructions. Overall, 58% of the trainees and 33% of the officers failed to notice a gun positioned in full view on the passenger dashboard. The driver’s style of interaction had little effect on noticing rates for either group. People can experience inattentional blindness for a potentially dangerous object in a naturalistic real-world context, even when noticing that object would change how they perform their primary task and even when their training focuses on awareness of potential threats.

## Significance

Real-world failures of awareness abound—in domains ranging from driving to radiology, people often fail to notice unexpected events when they are focusing their attention on something else (inattentional blindness). With few exceptions, inattentional blindness has been studied in a laboratory context using computerized presentations and videos, and most such studies examine the detection of salient, but task-irrelevant unexpected objects. Although the laboratory results appear consistent with descriptions of awareness failures in the world, most consequential real-world cases involve important and relevant objects rather than task-irrelevant ones.

This study examined whether experienced police officers and trainees at a police academy would notice a gun positioned in plain sight during a simulated vehicle stop. During a real vehicle stop, the presence of a gun would have direct and immediate consequences for the officer—it is relevant to their task. Moreover, police are trained to look for dangers in their environment. In our study, many trainees and officers failed to notice the gun. The driver’s interaction style during the vehicle stop (cooperative or agitated) had little effect on noticing rates. This approach allowed for a systematic study of noticing rates using a controlled simulation that mimics common reports of real-world inattentional blindness for relevant and potentially important objects.

## Background

People often fail to notice unexpected events when they are focusing attention on something else, a phenomenon known as inattentional blindness (Mack & Rock, 1998). Most studies of inattentional blindness manipulate attention using videos or simple computer displays; people focus attention on an arbitrary task—counting passes of basketballs, counting bounces of shapes, judging lengths of lines, etc.—and fail to notice unexpected objects or shapes passing through the display (e.g., Mack & Rock, 1998; Most et al., 2001; Simons & Chabris, 1999). Typically, the unexpected objects and their behaviors are salient and distinctive, but unrelated to the task and unimportant to the participant. In contrast, many real-world analogues of inattentional blindness involve unexpected objects with practical consequences: texting pedestrians accidentally walk into things (https://www.youtube.com/watch?v=lRYv_2JRCT0), drivers obliviously follow flawed GPS instructions (Hansen, 2013), or more commonly, car drivers fail to yield the right of way to motorcycles, leading to “looked but failed to see” accidents (see Hyman, 2016 for other examples). Only a handful of studies have examined inattentional blindness beyond the laboratory, and even fewer have examined noticing of task-relevant, naturalistic unexpected events that involve direct risks to and consequences for the attentively blinded.

Some studies have examined inattentional blindness using realistic, naturalistic tasks. Others have examined unexpected objects that are relevant to how people perform the primary task, and still others have examined unexpected objects that might be salient, relevant, or threatening for a participant. Ours is the first large-scale study to combine all of these common elements of real-world analogues of inattentional blindness.

Most studies using tasks that are relevant to participants have tested noticing of unexpected objects that were unusual or irrelevant to that task. For example, expert radiologists scanning through a sequence of x-ray images often failed to notice the superimposition of a small image of a gorilla on the radiograph (Drew, Võ, & Wolfe, 2013). The primary task—scanning through radiographs—was more relevant for them than the typical ball-counting tasks, but the unexpected object itself was unusual and had no direct consequence for the participants. Missing it would not result in a malpractice claim, for example. Similarly, experienced basketball players are somewhat more likely to notice an unexpected gorilla when counting passes (Memmert, 2006), perhaps because of their greater experience with something akin to the primary task. Again, though, the gorilla is irrelevant to how they perform that task. That experts can miss irrelevant unexpected objects when tested using familiar tasks is important, but it does not directly test whether that experience overrides inattentional blindness for a consequential unexpected object.

Other studies have examined unexpected objects that might be salient or personally relevant, but they have typically done so using somewhat arbitrary and artificial laboratory tasks. For example, while judging which of two lines is longer, people were more likely to notice a briefly flashed schematic smiley face than a scrambled face (Mack & Rock, 1998), and in the same line-judgment task, participants were more likely to notice and identify an unexpectedly flashed spider (posited to be evolutionarily relevant) than an irrelevant one like a housefly (New & German, 2015; note that they missed the objects at similar rates but were better able to localize and identify the spider when they saw it). Although some threatening and personally relevant stimuli might be noticed and identified at higher rates, these stimuli again are irrelevant to the primary task. And, the primary task is arbitrary and unlike real-world analogues of inattentional blindness.

A conceptually similar study examined whether an object associated with task-relevant risks or rewards would capture attention and be noticed (Stothart, Wright, Simons, & Boot, 2017). Participants played a simple computer game in which they dodged missiles fired by colored rectangles. Crucially, the damage from a missile depended on its color. After 8 minutes of playing this game, participants associated each color with greater or lesser consequences, and successfully avoided more of the costly missiles. Yet, noticing rates for unexpected objects were relatively unaffected by that object’s color: Noticing was no greater for objects that had the same color as the more consequential colored rectangles. Thus, even when the unexpected object’s color was associated with task-relevant judgments, it did not differentially draw attention. Again, the task was somewhat arbitrary and un-naturalistic, and the unexpected object did not directly affect how people performed the primary task.

Several studies have examined the detection of objects that were relevant to how people performed the primary task but that were still unexpected (Pammer, Bairnsfather, Burns, & Hellsing, 2015). When asked to judge whether briefly presented photographs depicted safe or unsafe driving scenes, many people failed to notice pedestrians positioned unexpectedly near the road, and they were somewhat more likely to notice pedestrians who constituted more of a hazard. Although this study used photographs of scenes, the task itself is not particularly naturalistic and experiential, and the unexpected objects were not directly consequential for the participants.

Similarly, when participants watch short videos of team handball or basketball, they often fail to notice an open player, even when they have to decide who should receive the next pass from the player holding the ball (Memmert & Furley, 2007; Furley, Memmert, & Heller, 2010). The unexpected object in this case is directly relevant to the participant’s task, but making judgments while watching a video differs from performing them in the world. Also, these studies focused on giving participants explicit instructions about how to attend and when to make their decision, making the task itself somewhat less naturalistic than playing in an actual game would be.

A few observational studies have examined inattentional blindness in the real world using salient unexpected objects, but most of those objects were irrelevant to the task or not particularly important for the participant. For example, participants walking down a sidewalk on a college campus can fail to notice a unicycling clown (Hyman, Boss, Wise, McKenzie, & Caggiano, 2010). Other observational studies have used task-relevant unexpected objects in the real world, but not objects that are threatening to the participant. For example, pedestrians avoid an obstacle along their path without realizing they have done so, and they bypass money hanging from a tree (Hyman, Sarb, & Wise-Swanson, 2014).

Inspired by a real criminal case in which a police officer claimed not to have noticed a fight occurring near him (see Chabris & Simons, 2010; Lehr, 2009), one study did examine inattentional blindness for a potentially consequential real-world event (Chabris, Weinberger, Fontaine, & Simons, 2011). Participants were asked to follow an experimenter at an easy jogging pace around a college campus and monitor how often the experimenter touched his hat (ensuring that they focused attention on the experimenter). Off to the side of the path, two people simulated an attack on a third person, hitting and kicking him. When the study was conducted at night, mimicking the criminal case, 65% of participants missed the fight. And, even in full daylight, 44 percent missed it. We might expect that participants who noticed a fight would call attention to it, but none of the participants stopped running to assist the person being attacked. Although the fight was salient and important, it presented no direct risk to them or consequences for them and it did not fundamentally change their primary task (jogging behind the experimenter).

Two questions remain unclear from these and earlier studies of inattentional blindness. First, when performing a familiar and naturalistic task, will people be more likely to notice an unexpected event that poses a direct risk to them, one that interferes with their ability to perform the task? Second, would they be more likely to detect it if, upon noticing, they would have to change their actions? That is, would they be more likely to notice unexpected events that, by their nature, change the demands of the primary task?

Other than the team-sports studies discussed above, only two other published studies have examined inattentional blindness in naturalistic tasks for objects/events that, if noticed, would require a change in the participant’s actions. In one early study, four commercial airline pilots participating in an extensive simulator training program completed a number of simulated landings while using a head-up display (Haines, 1991). On a critical trial, they emerged under a low cloud ceiling and another jet was sitting on the runway. Although it filled much of the cockpit window, two of the pilots never saw the plane and landed anyway, and the other two pilots intended to abort the landing but did so too late, resulting in a stopped trial. The study constitutes an existence proof that people can miss consequential task-relevant objects in a naturalistic simulation, but it included too few participants to estimate how often such failures occur or to examine any of the factors that might contribute to noticing.

The other study tested whether drivers would notice an unexpected motorcycle (Most & Astur, 2007) when using a laptop-based driving simulator. The study was primarily designed to examine the role of attention set for color: Participants responded to colored signals telling them which way to turn, and the motorcycle either shared the attended signal or matched an ignored signal color. When the unexpected motorcycle matched the attended color, only 7% of drivers collided with it. When it matched the ignored color, 36% of drivers collided with it. Two of those participants failed to apply the brakes at all, suggesting that they never noticed it.

Both of these studies examined unexpected events in a simulator context, with participants performing multiple trials of the task without any unexpected objects. They suggest that people can miss consequential, task-relevant unexpected objects that we would expect to influence their performance on the task. But, no studies have examined such failures in a real-world context during the course of a single, naturalistic, familiar task.

We examined whether inattentional blindness would occur under naturalistic conditions in a familiar task for an object that, if noticed, should immediately alter the nature of that task. Specifically, we tested whether police officers and police academy trainees engaged in a simulated vehicle stop would notice a handgun placed in plain sight on the passenger dashboard of a stopped car. A visible gun in a car poses a direct risk to the officer, and when a patrol officer notices a gun in a car, the nature of the interaction immediately changes. They can take a wide variety of steps, ranging from drawing their own weapon if they believe they are in imminent danger to calmly noting the presence of a gun to the driver and asking if they have a permit. But, regardless of their response, they call attention to the presence of the gun as soon as they see it.

If threatening or high-risk objects in plain sight draw attention, officers should be likely to see it and call attention to it. That is, the potential threat should override inattentional blindness. Alternatively, if unexpected objects are only processed after they reach conscious awareness, then the implied threat might have little effect on conscious noticing, and officers will have inattentional blindness for the gun. We address whether inattentional blindness occurs during this naturalistic, commonly performed task with an unexpected object of direct relevance to the participant. Although anecdotal evidence from real-world analogues suggests that inattentional blindness is common (e.g., in distracted walking or driving), no studies have demonstrated such inattentional blindness under controlled conditions.

As part of the study design, we also manipulated whether the driver’s reaction to being stopped (passive and cooperative versus angry and agitated) would affected the likelihood that participants would notice the gun. We might predict less noticing when the driver is agitated because the officer would be more likely to narrow their attention to the driver. Alternatively, with an agitated driver, an officer might judge the situation to be riskier, leading them to pay more attention to potential threats; if potential risks attract attention, highlighting the threatening nature of the situation could increase noticing.

## Methods

### Preregistration and departures from the plan

This study was preregistered, and the plans, data scripts, and data are publicly available (https://osf.io/gkt84/). Note that the publicly available data omit the age, sex, and patrol experience demographic information as that information could be used to re-identify participants. A complete dataset including the demographic data is available to qualified researchers upon request.

The procedures for the two studies were identical, with study 1 focusing on trainees at the Police Training Institute (a police academy) and study 2 focusing on experienced patrol officers returning to the Police Training Institute for refresher courses. Although testing sessions for these groups of participants occurred over the same time period, each session included participants from only one group. Our implementation in study 2 deviated from our preregistered plan in several respects, mostly due to unexpected challenges in recruiting experienced officers. First, fewer experienced officers attended the sessions than expected, so we needed more sessions to complete the study. We also stopped testing with 75 experienced officers rather than our specified target of 80 because we were unable to schedule additional sessions with experienced officers in a timely fashion. That decision was made before tallying the results from the final session. In total, data collection took place over the course of 3 years (rather than the anticipated 1 year). In all other respects, testing followed the preregistered plan. Although the studies with trainees and with experienced officers were preregistered separately, we describe their methods and results jointly given that they used identical procedures.

### Setting

The Illinois Police Training Institute (http://pti.illinois.edu/about/index.html) provides a 480-hour basic police training academy. The trainees are not yet “certified” police officers, but they have been hired by an Illinois police department and sent to the Police Training Institute for training. They are taught how to conduct a vehicle stop, how to defuse a tense situation, how to respond to threats, etc. The 12-week program includes extensive, hands-on practice in simulated interactions with civilians under naturalistic and realistic conditions. In addition to serving as a police academy for trainees, the Police Training Institute also holds 1-day “refresher sessions” for experienced officers in which they discuss updates to standard practices, engage in scenario simulations, and discuss differences in how their home departments handle common situations.

As part of their police academy training, trainees perform a number of simulated vehicle stops in which an experienced actor plays the role of the driver. The trainees are informed about the type of stop they are making (e.g., suspect in a crime, stopping a vehicle of a crime suspect, vehicle stops with unknown risk such as failing to stop at a stop sign, etc.), and the scenarios vary in intensity, from routine to highly threatening. They are designed to teach strategy and tactics, including control and arrest, suspect management, use of force decision making, approach and deployment, non-escalation techniques, and de-escalation techniques.

The trainees in our study were in the fifth or sixth week of the program, had received classroom instruction on vehicle stops, and had participated in 4–8 hours of vehicle stop scenarios. The role player in our study has served as an actor in these training simulations for years. He is practiced at simulating a cooperative driver as well as an agitated/hostile one.

### Participants

Participants included 100 police trainees (94 male, 6 female) and 75 experienced police officers (67 male, 8 female). One additional experienced officer declined to answer questions after the car-stop event. Some of the trainees had previous experience working as guards in the prison system or as military police, and a few had participated in “ride alongs” with experienced patrol officers, but none had patrol experience of their own or had conducted real vehicle stops. The experienced officers reported an average of 12.3 years (SD = 8.0) of patrol experience, and they were somewhat older (M = 38.4 years, SD = 9.4) than the trainees (M = 26.7 years, SD = 5.3).

### Procedure

Participants in the study engaged in a routine vehicle stop scenario in which a gun was unexpectedly visible in the car. The primary question was whether people would notice the gun.

**Fig. 1:**
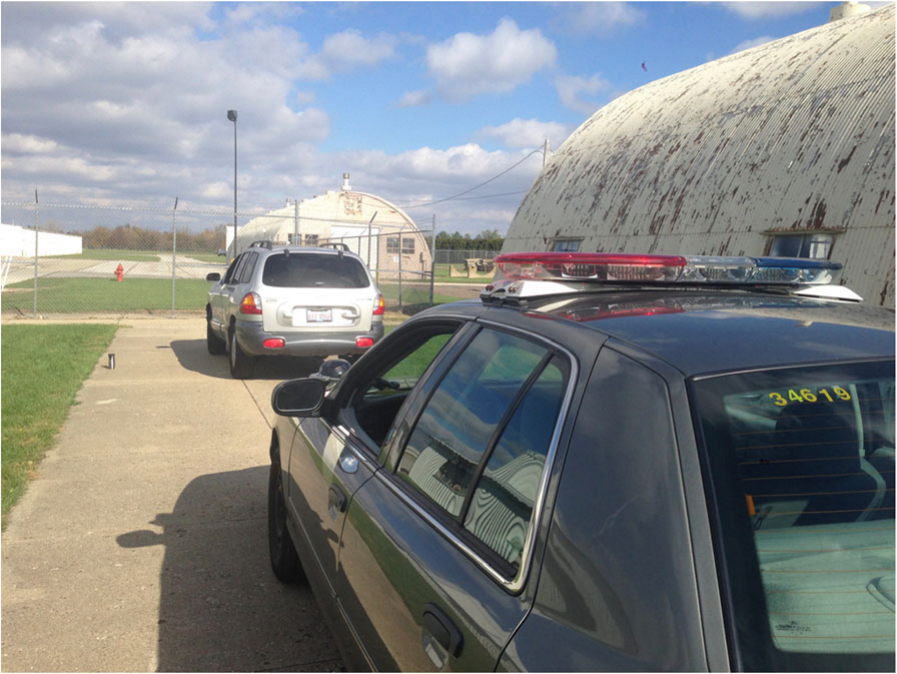
Image of the police car stopped behind the pedestrian’s car in the vehicle stop scenario

Participants in the study were excused from a classroom setting or other simulation and asked to go see Dr. Schlosser (who is the Director of the Police Training Institute) to complete another scenario. Dr. Schlosser asked the participant to remove their standard duty belt (in case they were carrying a loaded gun) and to replace it with a duty belt with a safe-training, inoperable handgun. People attending the Institute are used to completing scenarios as part of their regular training and they commonly switch to a different belt and training handgun to avoid any risk of an accidental shooting. Once the participant was ready, Dr. Schlosser explained that they had pulled over a vehicle because the driver failed to stop at a stop sign. He instructed them to use their discretion to decide whether to issue a traffic citation or a warning citation. An actor sat in a parked car positioned between two buildings and sheltered from view from other people at the Institute, about 10 m from where the participant met Dr. Schlosser (Fig. 1).

The participant approached the car and engaged with the driver using their standard procedures for that situation. Dr. Schlosser monitored the interaction from a position about 3–5 m behind the officer. The “driver” responded to the approaching officer in one of two ways. In the “compliant” condition, the driver admitted fault and was apologetic, polite, and friendly. He immediately and appropriately complied with all requests. In the “aggressive” condition, the driver was verbally hostile, agitated, and overtly upset. He complained about unfair treatment and implied that the officer pulled him over to fulfill a quota. Although he acted upset, he complied with all instructions (i.e., he provided his license and registration when asked).

Participants were assigned to conditions in a fixed random order (see preregistration), and the same random order was used for both the trainee sample and the experienced officer sample. The random order was created such that there were exactly 40 participants assigned to each condition in each block of 80 participants. Because one condition had 75 participants and the other had 100, neither group of participants had exactly equal numbers of people assigned to the two conditions (see Table 1 for sample sizes, noticing rates, and comparisons across groups). Dr. Schlosser remained blind to the testing condition until the driver began interacting with the participant.

**Table 1:** Noticing rates and statistical difference as a function of participant group and driver response

	Officers	Trainees	Combined	Officers–trainees
Compliant	71.1% (27/38)	45.1% (23/51)	56.2% (50/89)	26% Z = 2.44, *p* = 0.015
Aggressive	62.2% (23/37)	38.8% (19/49)	48.8% (42/86)	23.4% Z = 2.15, *p* = 0.032
Combined	66.7% (50/75)	42% (42/100)	52.6% (92/175)	24.7% Z = 3.23, *p* = 0.001
Compliant–aggressive	8.9% (Z = 0.82, *p* = 0.414)	6.3% (Z = 0.64, *p* = 0.522)	7.4% (Z = −0.92, *p* = 0.331)	

Z = (Proportion1 − Proportion 2) / Sqrt([Combined proportion] * [1-Combined proportion] * [1/n1 + 1/n2]). For example, the upper-right Z score = (0.771 − 0.451) / Sqrt([0.562][1 − 0.562][1/38 + 1/51]). The *p* values are two-tailed

**Fig. 2:**
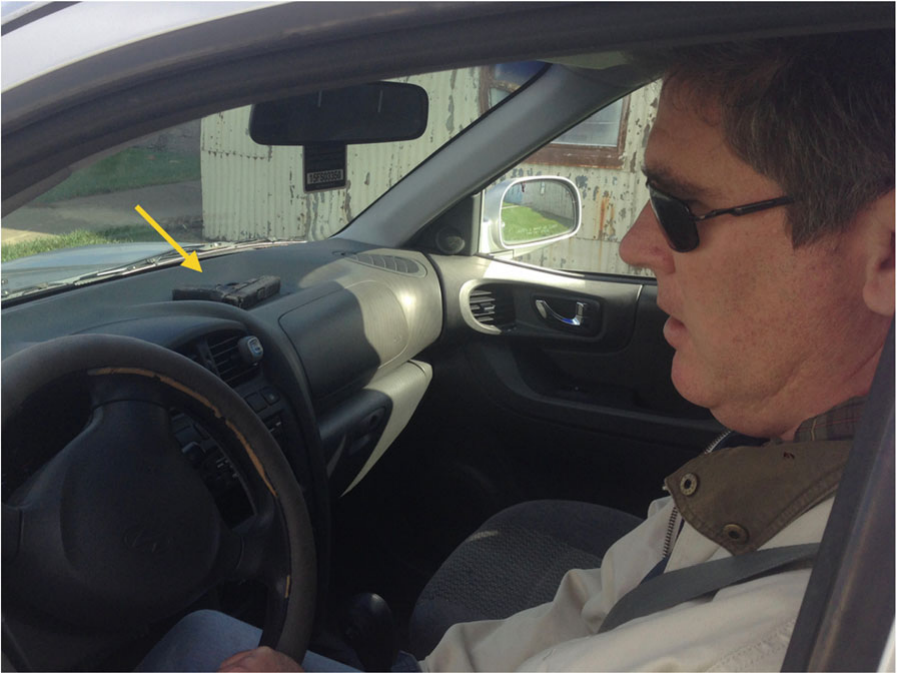
View through the driver-side window, with a gun visible on the passenger dash (indicated by the yellow arrow)

In addition to typical vehicle contents, we positioned an unloaded airsoft pistol on the dashboard above the glovebox so that it would be fully visible to the participant through the driver’s window (Fig. 2). Given the nature of the unexpected object and the requirements of police procedure, it was clear from their actions during the simulated vehicle stop whether or not they had noticed the gun. When participants noticed the gun, they always called attention to it and took appropriate measures (ranging from discussing it with the driver to drawing their own weapon and instructing the driver to exit the vehicle—officers have discretion about how to handle the situation). In such cases, Schlosser interrupted the interaction and directed the participant to Simons who was waiting out of sight in a neighboring building where he conducted the post-scenario questioning. If the officer did not notice the gun, they typically completed the vehicle stop simulation without interruption before being directed to Simons.

Simons then explained that we were conducting a study, and asked the officer if they would be willing to answer a few questions about the vehicle stop. If they were (one officer declined), they provided written informed consent and Simons then read the following questions and manually recorded their responses:

1. “During the traffic stop, did you notice anything that might have been a danger to you?”
2. “Did you notice any weapons?”
3. “Did you notice any drug paraphernalia?”
4. “Was the driver aggressive in any way?”
5. “In a real traffic stop, would you have felt in danger from a driver like the one you stopped?”
6. “Have you heard about this particular traffic stop scenario [from other trainees | from anyone else]?”
7. “How old are you?”
8. Trainees: “Do you have any patrol experience?” Officers: “How long have you been on the force?”

Participants were asked to explain any “yes” response and Simons summarized their responses on the form. If they spontaneously mentioned a gun in response to the question about noticing a weapon (which they did whenever they had noticed it), we did not ask the second question about noticing a gun (we coded their answer as “yes”). There were no drug paraphernalia in the car—that question was intended to catch people who were biased to say “yes” when asked about anything they had not seen. No participant falsely reported drug paraphernalia. All participants reported that they had not heard any details about this scenario from any of the other participants at the Police Training Institute.

## Results

We examined whether the percentage of people noticing the gun differed when the actor was compliant or aggressive and whether trainees and experienced officers differed in their noticing rates. Our preregistered analysis consisted of two-tailed Z tests of the difference in proportions of participants noticing the gun (for condition, subject group, and condition separately for each subject group; see Table 1).

Overall, only 52.6% of participants noticed the gun even though it was fully visible on the passenger dashboard throughout the interaction between the driver and the police officer. Many of those who missed the gun expressed surprise or chagrin that they had missed it when later given the opportunity to view the car again. Experienced officers were substantially more likely to notice the gun (66.7%) than were trainees (42%), but 1/3 of them missed the gun as well.

Noticing was minimally affected by whether the driver was aggressive or compliant. A slightly larger proportion of both trainees and experienced officers noticed the gun in the compliant condition than in the aggressive condition, but the difference between conditions was small, not statistically significant, and roughly comparable for trainees and officers.

Our preregistration specified an exploratory analysis of the contributions to noticing of sex and age differences and patrol experience (with no a priori predictions). Too few of the participants were female to justify exploring sex differences, and the vast majority of the trainees were of similar age (M = 26.7, SD = 5.3 years) and had little or no patrol experience. For the experienced officers, neither patrol experience (*r* = .08) nor age (*r* = .005) was meaningfully associated with noticing.

## Discussion

When completing a routine vehicle stop scenario, both police trainees and experienced patrol officers frequently failed to notice a gun placed conspicuously on the passenger dashboard. Although experienced officers were more likely than trainees to notice the gun, 1/3 of them missed it and proceeded to cite the driver. This study provides clear evidence that experts performing a naturalistic task in their domain of expertise can miss a potentially dangerous unexpected object that would have direct consequences for them and the way they perform their task. Moreover, this failure of awareness occurred for a group of participants (police officers) trained to look for and assess threats.

Although many trainees and experienced officers missed the gun, noticing rates were relatively unaffected by whether the driver responded in a passive/compliant manner or an aggressive/hostile one. Drivers in both groups were slightly more likely to notice the gun when the driver was calm, but the differences were small and could plausibly be attributed to chance. This pattern is inconsistent with the idea that people will be more likely to notice threatening unexpected objects in contexts that are more stressful or potentially more dangerous.

Overall, experienced officers were more likely to notice the gun than were trainees, and anecdotally, many noticed it early in the interaction, often before asking for the driver’s license and registration. Experience might allow the officers to inspect the car more efficiently at the start of their standard patter, giving them greater situation awareness. Alternatively, greater noticing rates could result from their fluency with standard vehicle stop procedures; experienced officers likely do not need to devote much attention to what to say, where to stand, and what steps to take in response to the driver’s comments. Unlike experienced officers, trainees are still learning the guidelines for vehicle stops and might devote more attention to those procedures than to the situation or the contents of the car. This difference in experience with the procedures might explain the lower rates of noticing for trainees without any need to appeal to greater efficiency in searching the car or inherently better situation awareness.

The lack of a difference between trainees and experienced officers as a function of the driver’s attitude was somewhat surprising. Unlike trainees, experienced patrol officers have encountered many agitated, angry civilians during their years of daily police work. We expected that they would be less flustered by an angry driver than would trainees. Yet, both groups showed little difference in noticing as a result of the driver’s attitude. The lack of a difference could mean that the nature of the interaction has little bearing on what police notice or it could mean that something about the situation mitigated the effect of the driver’s behavior. For example, the difference in the actor’s reactions might have not have been dramatic enough (although most of the officers noted that he was agitated and upset in the “aggressive” condition). Or, the knowledge that it was a simulation could have reduced the potential stressfulness of the encounter; the scenario used in this study might not have been stressful enough for the participants to observe differences in the rate of noting unexpected objects. Finally, in some cases, participants noticed the gun early in the interaction, minimizing their exposure to the driver’s reaction. Those cases might dilute any difference between the conditions.

### Constraints on generality

This study provides evidence that both police academy trainees and experienced officers can miss an unexpected gun in a simulated vehicle stop. Although we expect that officers conducting a real vehicle stop would also experience inattentional blindness, it is possible that differences between a real stop and our simulated one could increase or decrease the rate of noticing. Given that our study was conducted at a police academy with the academy’s director observing the interaction, participants might have been more focused on their vehicle stop procedures than they would be when actually patrolling. They also knew that they were not in danger from the simulation, despite its realism. None of the participants expressed suspicion that there would be an unexpected object, but some might have treated the scenario as a test rather than a vehicle stop, and that could have affected how they focused attention during the interaction.

The position of the gun in our study was somewhat unusual—few people leave a gun sitting on the passenger dashboard. And, the rarity of seeing a gun in that location might contribute to a failure to notice it (see Wolfe, Horowitz, & Kenner, 2005 for studies of how rarity affects detection in a deliberate search by airport baggage screeners). Noticing rates might differ if the gun were kept in a more typical location, but using a more typical location would have made studying inattentional blindness in this context impossible—most people who carry a gun in their car keep it hidden (e.g., in the glove box). If the unusual location of the gun decreased noticing rates, we would expect the odd location of the gun to have a bigger effect for experienced officers because they would have stronger expectations about the likeliest locations for a gun. If so, the location of the gun might have amplified the difference in noticing rates between experienced officers and trainees. However, the fact that many experienced officers detected the gun rapidly suggests that the oddness of the location did not substantially reduce noticing.

Our observed lack of a difference between the compliant and aggressive driver conditions might not generalize to a real stop. Although the driver was an experienced actor and effectively conveyed a cooperative/agitated demeanor, participants might have been less affected by the difference in reactions than they would have been during a real vehicle stop.

Given that the participants came from a wide range of jurisdictions, some with higher rates of violent crime than others, we would expect the pattern of results to hold for trainees and experienced officers from most jurisdictions in the USA (where guns are relatively common and police practices for vehicle stops are fairly standard). The participants in our study were mostly white men, and we do not know if the pattern of results would be consistent with participants from other demographic groups. Moreover, it is possible the pattern of noticing would differ if the actor were female and/or non-white. Other than these factors, we have no reason to believe that the results depend on other characteristics of the participants, materials, or context.

## Conclusion

Participants can fail to notice an unexpected object even when that object presents a potential threat and is relevant to how they perform their primary task. Police officers and trainees often failed to notice a gun in full view despite the emphasis in police work on vigilance for possible dangers and threats. To our knowledge, this study is the first to demonstrate robust inattentional blindness for an object of direct relevance to participants and their actions in a naturalistic situation.

In this study, the driver’s demeanor had little effect on noticing rates. The nature of the interaction might have a greater effect during a real traffic stop, and the small difference in noticing that we did observe might prove to be robust with a substantially larger sample. The size of that effect and the rates of inattentional blindness might also interact with other unexplored factors, including the race/sex of the driver. If the results do generalize to real vehicle stops and if they hold true for other demographic groups, they could lead to better police training. Most officers who experienced inattentional blindness in our study were surprised that they could have missed the gun—like other participants in inattentional blindness studies, they expected that they would automatically notice something salient and relevant (Levin & Angelone, 2008). Police training could focus on dispelling that misconception, and it could also highlight how the nature of the interaction does not strongly predict whether or not an officer will notice an unexpected threat.
